# Optimising an intervention to support home-living older adults at risk of malnutrition: a qualitative study

**DOI:** 10.1186/s12875-021-01572-z

**Published:** 2021-11-11

**Authors:** Liz Payne, Daniela Ghio, Elisabeth Grey, Joanna Slodkowska-Barabasz, Philine Harris, Michelle Sutcliffe, Sue Green, Helen C. Roberts, Caroline Childs, Sian Robinson, Bernard Gudgin, Pam Holloway, Jo Kelly, Kathy Wallis, Oliver Dean, Paul Aveyard, Paramjit Gill, Mike Stroud, Paul Little, Lucy Yardley, Leanne Morrison

**Affiliations:** 1 School of Psychology, University of Southampton, Southampton, UK; 2 Primary Care Population Sciences and Medical Education, University of Southampton, Southampton, UK; 3 Department for Health, University of Bath, Bath, UK; 4 Dietetics Department, University Hospital Southampton NHS Foundation Trust, Southampton, UK; 5 Department for Nursing Science, Bournemouth University, Poole, UK; 6 Academic Geriatric Medicine, Faculty of Medicine, University of Southampton, Southampton, UK; 7 Human Development and Health, University of Southampton, Southampton, UK; 8 AGE Research Group, Translational and Clinical Research Institute, Newcastle University, Newcastle upon Tyne, UK; 9 NIHR Newcastle Biomedical Research Centre, Newcastle University and Newcastle upon Tyne Hospitals NHS Foundation Trust, Newcastle upon Tyne, UK; 10 Public and Patient Involvement, University of Southampton, Southampton, UK; 11 Wessex Academic Health Science Network, Southampton, UK; 12 Faculty of Medicine, University of Southampton, Southampton, UK; 13 Nuffield Department of Primary Care Health Sciences, University of Oxford, Oxford, UK; 14 Warwick Medical School, University of Warwick, Coventry, UK; 15 Clinical Nutrition, University of Southampton, Southampton, UK; 16 School of Psychological Science, University of Bristol, Bristol, UK

**Keywords:** Person-based approach, Malnutrition, Eating patterns, Intervention planning, Ageing; primary health care, General practice, Independent living, Health services for the aged, Dietary supplements

## Abstract

**Background:**

In the UK, about 14% of community-dwelling adults aged 65 and over are estimated to be at risk of malnutrition. Screening older adults in primary care and treating those at risk may help to reduce malnutrition risk, reduce the resulting need for healthcare use and improve quality of life. Interventions are needed to raise older adults’ risk awareness, offer relevant and meaningful strategies to address risk and support general practices to deliver treatment and support.

**Methods:**

Using the Person-based Approach and input from Patient and Public Involvement representatives, we developed the ‘Eat well, feel well, stay well’ intervention. The intervention was optimised using qualitative data from think aloud and semi-structured process evaluation interviews with 23 and 18 older adults respectively. Positive and negative comments were extracted to inform rapid iterative modifications to support engagement with the intervention. Data were then analysed thematically and final adjustments made, to optimise the meaningfulness of the intervention for the target population.

**Results:**

Participants’ comments were generally positive*.* This paper focuses predominantly on participants’ negative reactions, to illustrate the changes needed to ensure that intervention materials were optimally relevant and meaningful to older adults. Key factors that undermined engagement included: resistance to the recommended nutritional intake among those with reduced appetite or eating difficulties, particularly frequent eating and high energy options; reluctance to gain weight; and a perception that advice did not align with participants’ specific personal preferences and eating difficulties. We addressed these issues by adjusting the communication of eating goals to be more closely aligned with older adults’ beliefs about good nutrition, and acceptable and feasible eating patterns. We also adjusted the suggested tips and strategies to fit better with older adults’ everyday activities, values and beliefs.

**Conclusions:**

Using iterative qualitative methods facilitated the identification of key behavioural and contextual elements that supported engagement, and issues that undermined older adults’ engagement with intervention content. This informed crucial revisions to the intervention content that enabled us to maximise the meaningfulness, relevance and feasibility of the key messages and suggested strategies to address malnutrition risk, and therefore optimise engagement with the intervention and the behavioural advice it provided.

**Supplementary Information:**

The online version contains supplementary material available at 10.1186/s12875-021-01572-z.

## Background

In the UK, 1.3 million adults (11%) aged 65 and over are estimated to be malnourished [1, 2], with over 5500 episodes of primary or secondary malnutrition among adults aged 60 and over admitted to NHS hospitals in England annually [3]. Around 14% of community-dwelling older adults are estimated to be at risk of malnutrition, rising to 18% of those receiving day care and home care [1]. It is suggested that most malnutrition develops in the community through ongoing undernutrition of which individuals may be unaware [4]. Half of the estimated £20 billion cost of health and social care for malnutrition in 2011–12 was for older adults, most of which was for institutional care or hospitalisation [1].

Malnutrition diagnosis is based on objective measures of weight, muscle strength, food intake, inflammation and disease burden [5]. Malnutrition risk measured by tools such as Malnutrition Universal Screening Tool (MUST) [6] or the Mini Nutritional Assessment (MNA) [7] is associated with frailty and sarcopenia [8], falls [9, 10], more GP consultations and hospitalisation [11, 12] and reduced quality of life [13], particularly among those needing support from meal services and social services [14]. Addressing malnutrition or malnutrition risk, which undermines the immune system, is also essential to protect older adults against poor outcomes should they contract novel coronavirus covid19 (C19), [15].

Identifying those at risk in primary care, and offering treatment, may reduce healthcare use and mortality risk, and improve health and quality of life [1, 12, 16–18]. Screening tools such as MUST or MNA provide an early assessment of malnutrition risk, and some also identify the health or social factors that contribute to greater nutritional risk, such as appetite loss, depression or immobility [7], chewing problems, eating alone or difficulty with food shopping [19]. Indeed, existing community interventions have provided advice about adapting food textures, dental referral for chewing, swallowing and oral health problems [20], personalised home care to encourage three daily snacks, action plans, tips addressing eating difficulties, and referral to health professionals and local initiatives [21] and nutritional education for carers [22]. Some of these interventions have shown promise, reporting improved mental functioning, nutritional intake, nutritional knowledge and/or risk scores [20–22]. However, this is an under-researched area given the magnitude of the problem and the wide range of risk factors, and previous studies have not examined the impact of early intervention on quality of life (QoL).

To optimise the effectiveness of community interventions in reducing malnutrition risk we first need to ensure that the advice and behavioural support provided is meaningful to participants. This is challenging given that there are multiple barriers to following advice, particularly psychosocial barriers [23, 24], which can undermine motivation to change eating behaviours, even when practical and physiological support is given [23–25]. Older adults may not accept that they are undernourished [26–29], or relate to the term ‘malnutrition’, which can induce fear or offence, perhaps deterring healthcare professionals from discussing it [30]. Additionally, older adults may not recognise that nutrition is important [29, 31]; and promotion of general public health advice may contribute to the belief that weight loss is a normal or positive part of ageing [32]. Life changes such as bereavement [20, 33] or being alone [27] can contribute to apathy towards self-care [24]. Crucially, older adults can become resigned to beliefs about inevitable decline during ageing which undermines their motivation and confidence to make changes to improve nutritional self-care in the face of health problems and fears about loss of independence [24]. In the present study, we developed and optimised an intervention (Eat well, feel well, stay well) to address malnutrition risk among older adults with health or social conditions that may make them vulnerable to malnutrition.

### Aims

We aimed to identify beliefs or contextual issues that undermined or supported older adults’ engagement with a prototype intervention to address risk of malnutrition. The following question was addressed: What undermines or supports older adults to experience the intervention as useful, meaningful and engaging?

## Methods

### Design

We used the Person-Based Approach (PBA) to develop and optimise our intervention. The PBA is a systematic approach to applying qualitative research in intervention development [34], which seeks to understand individuals’ experiences and environment and address issues identified at every stage of intervention planning, development and testing (see Band et al., 2017 [35] for details). The aim is to ensure that interventions are highly relevant and meaningful for those who will use them while retaining theory and evidence-based elements supporting beneficial behaviour change [36]. Hence interventions are more likely to be used, perceived to be useful and effective.

An intervention for older adults at risk of malnutrition was optimised in parallel with carrying out ‘Think aloud’ interviews [37]. Process evaluation interviews were subsequently carried out during a feasibility study, after which the intervention was adjusted further. The methods used were pragmatic, to ensure timely delivery of an optimised intervention ready for trial. The study is reported following COREQ criteria [38].

### Prototype intervention

**Fig. 1:**
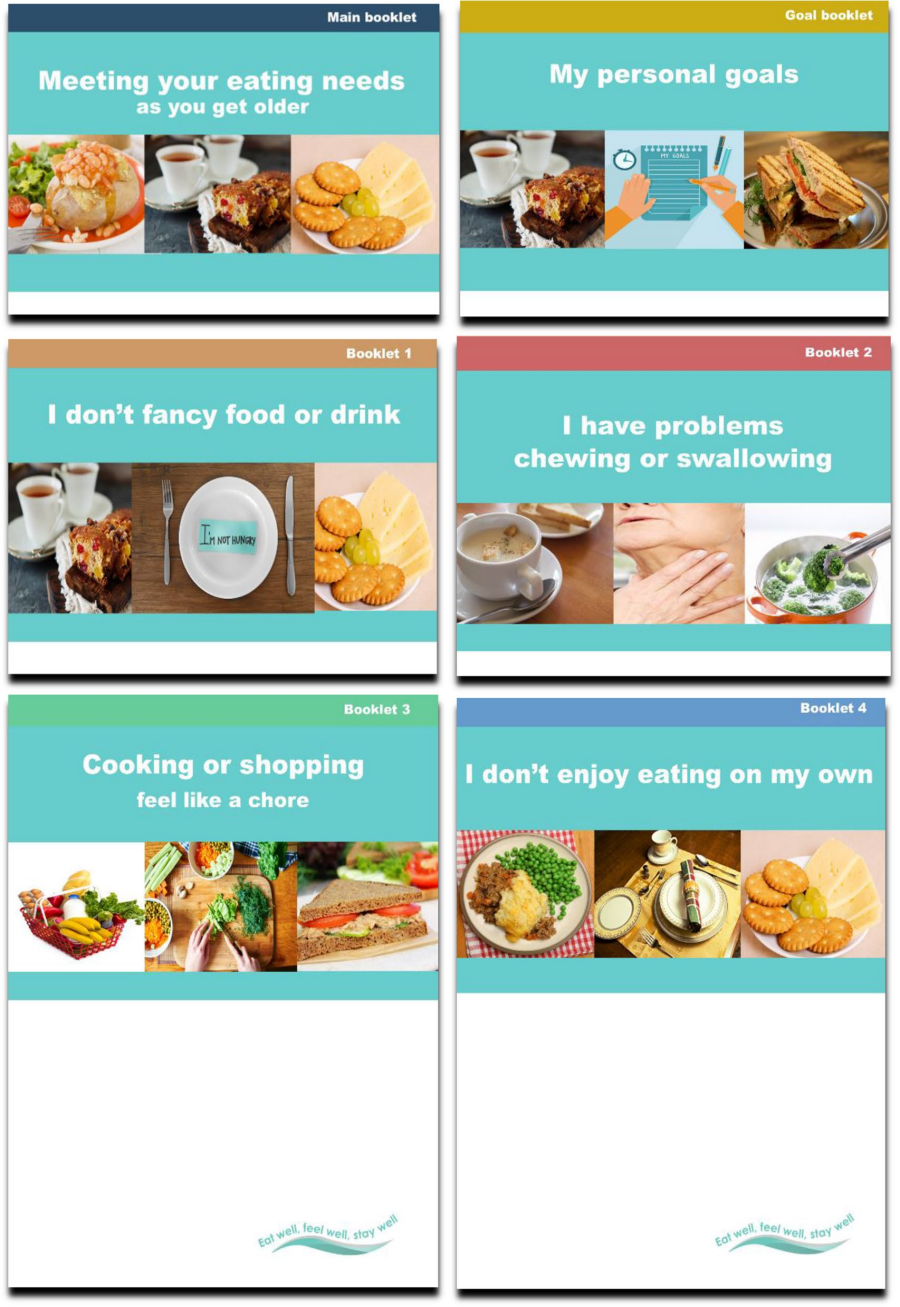
Eat well, feel well, stay well: Booklets

A prototype intervention was developed based on findings from our mixed methods synthesis [23], and exploratory interviews with older adults with malnutrition risk factors [24]. The prototype comprised a series of booklets, a food list and goal cards for patients (Fig. 1).

The materials were designed by a team of intervention development researchers with different levels of expertise (LP;DG;EG;JSB;PH;LM), with input from experienced dietitians, nutritionists and clinicians (MSu;SG;HR;CC;SR;MSt;PL). They are aimed at older adults with a lower BMI with low appetite or unintentional weight loss. The materials were designed to be used with light touch support from a healthcare professional (e.g. as an aid to discussing how to address individuals’ particular challenges during brief consultations or phone calls) and to respect older adults’ needs and desire to retain independence (outlined in Payne 2020 [24]). In our feasibility study, the booklets were used in an initial 20 min nurse consultation, and up to four brief follow-up phone or in-person discussions over 6 months, depending on patient needs.

Elements from social cognitive theory and self-determination theory were incorporated to inform the design of specific intervention components, identified as crucial to address the key behavioural and contextual issues identified during intervention planning, e.g. techniques to enhance self-efficacy, autonomy, motivation (see Payne et al., 2020 [24]]. Specific components characterising these features are shown in Table 1. Examples of how these components were incorporated in the intervention are shown in (Additional file 1). Readability was not formally assessed, but we used brief, simple sentences and common words with few syllables wherever possible, and a standard layout, colours and fonts, to enhance readability for a wide range of people. The booklets were reviewed by our public contributors in addition to participants in these interview studies.

**Table 1:** Guiding principles for intervention development describing the key behavioural issues, design objectives and intervention features and components

Key behavioural issues	Design objective	Key intervention features and components
1) Denial of risk and low motivation to engage with lifestyle change: Older adults unaware or unwilling to acknowledge personal risk of malnutrition and its impact on health and wellbeing. Numerous physical and social barriers to eating well.	Motivate engagement with lifestyle change	• Provide credible evidence-based rationale • Dispel myth that decline in appetite and eating, and weight loss are normal and inevitable in older age • Clarify that everyone can be at risk of malnutrition • Outline that mainstream public health messages (e.g. low fat, low sugar) may be less appropriate for those with low appetite or unintended weight loss • Demonstrate empathy and acknowledge real barriers to changing eating behaviour
2) Low self-efficacy to overcome physical and social barriers to eating well and make long-term changes, particularly resignation to age-related decline	Promote self-efficacy to manage malnutrition risk, and overcome barriers to eating well	• Positive tone to encourage beliefs about being able to overcome barriers • Align behavioural advice and support with need to be ‘well’ and ‘independent’ • Provide examples of small, easy to enact lifestyle-compatible changes • Allow self-tailoring to address personal barriers e.g. strategies for those who dislike eating alone • Offer longitudinal motivational support e.g. ongoing nurse appointments to encourage self-care • Support motivation in-the-moment e.g. suggest using visual cues (biscuit tin near kettle) to encourage eating between meals • Support personal goal and action planning • Provide stories to model successful ways to overcome barriers
3) Rejection of imposed lifestyle change that contradicts existing knowledge, values and preferences, including desire to remain independent	Promote support and autonomy for choosing lifestyle changes that harness personally relevant motivations	• Present rationale for lifestyle change with a non-directive tone • Present behavioural suggestions as options to try • Invite expression of preferences e.g. possible reasons for wanting to eat well, with tick boxes for self-selection • Acknowledge and validate existing knowledge and experience before introducing new information and advice

### Participants

Twenty-six ‘Think aloud’ interviews were carried out with 23 participants: 21 were face-to-face, and five by telephone (decided according to participant preference), prior to the feasibility study. Eighteen face-to-face process evaluation interviews were conducted with participants taking part in the feasibility study (ISRCTN76863664 [39]). Participants were free-living adults aged 65 and over, meeting the following criteria which made them more vulnerable to risk of malnutrition:

- Chronic health conditions e.g. Chronic obstructive pulmonary disease (COPD), cerebrovascular disease; cardiac failure; chronic kidney disease (stage IIIb/IV/V); liver disorders; Parkinson’s disease; current depression, OR
- Hospital stay in the previous 6 months, OR
- Living alone.

Process evaluation participants additionally had at least one self-report or nurse-measured low appetite or malnutrition risk indicator (MUST positive, SNAQ (Simplified Nutritional Appetite Questionnaire) score < 14, unintended weight loss in the last 3 months).

### Procedure

Participants were identified via general practice database searches in South Central England, or by snowballing after sharing study details through word-of-mouth. The general practice sample was purposive, including men and women of different ages, living alone or with a partner. We anticipated needing to carry out around 25 Think aloud and 20 process evaluation interviews, based on prior experience of intervention development. Those interested in participating completed a reply slip after receiving a participant information sheet and consent form. Researchers phoned to confirm candidates were happy to participate and arranged interviews. Think aloud participants were asked: “How would you rate your overall health during the past week? On a score of 1 to 7, where 1 is very poor and 7 is very good”. Process evaluation participants were not asked this question.

Consent forms were signed at the start of face-to-face interviews, and signed and sent to researchers by phone participants. Carers or spouses could be present. Recruitment stopped once a range of views was given and the research team agreed that participants were expressing no new addressable issues. The ‘think aloud’ interviews took place between November 2016 and December 2017, and ‘Process evaluation’ interviews took place between June 2018 and November 2018. Interviews were audio-recorded.

The ‘think aloud’ interviews, lasting 20–90 min, were conducted in participants’ homes or by telephone. Intervention materials were taken to face-to-face interviews and sent to telephone participants two weeks prior to interview. Participants were asked to look through intervention materials and say out loud what they were thinking. This technique has been widely employed to test usability during digital intervention development [37], and for non-digital interventions, e.g. Pasterfield et al., 2019 [40]. Interviewers used neutral prompts based on an interview guide (Additional file 2). The ‘process evaluation’ interviews, lasting 22–66 min, were conducted in participants’ homes one-three months after the nurse appointments. Intervention materials were first introduced/chosen during the nurse appointments at the GP surgery, and taken home for use by participants. Interviewers used neutral prompts based on an interview guide and additionally probed how the materials were used based on a topic guide (Additional file 2).

### Analysis

Data were analysed in two phases. First, in order to rigorously and rapidly process interview data, the ‘Table of Changes’ (TOC) method was used to extract positive responses, but also to identify key issues that appeared to be undermining engagement. Data were analysed (byDG;PH;LP;LM;EG) after each batch of think-aloud interviews and iterative changes made to optimise the intervention ahead of the feasibility study. Qualitative process evaluation data from the feasibility study was also rapidly analysed using the TOC method. Second, a detailed reflexive thematic analysis of the same data was subsequently completed to ensure that all aspects of participants’ experiences of using the prototype intervention had been identified and issues addressed before the intervention was evaluated in a large RCT. A critical realist perspective was adopted, acknowledging that participants’ reported experiences of the intervention reflect a real world, influenced by participants’ psychosocial and practical context.

## Table of changes

Participant responses about intervention content guided rapid ongoing adjustments throughout intervention development and optimisation. The TOC method, adapted from Bradbury et al., 2018 [36], was used to tabulate participants’ positive and negative comments about intervention materials, record possible changes and reasons for these changes using a coding framework, and prioritise essential changes using MoSCoW criteria (Must have, Should have, Could have, Won’t have) [41]. Suggested changes were discussed by the development team (DG,LP,LM,PH,JSB,EG,LY), and co-authors with specific clinical (MSt,SG,HR,PA,PG), nutritional (CC,SR,MSu,SG,KW) or older age expertise (HR), including patient and public involvement representatives (BG,PHo), before implementation.

## Thematic analysis

Interviews were transcribed verbatim by a professional transcriber and analysed using inductive reflexive thematic analysis [42, 43]. The aim was to answer specific research questions to further intervention development and optimisation. This second analysis, carried out once the rapid TOC analysis was completed, was conducted to 1) enable richer interpretation of why particular elements might be supporting or undermining engagement, and 2) support the research team to reflect on whether the rapidly extracted TOC comments had been understood in the wider context of participants’ experiences and thus addressed in appropriate and meaningful ways in the intervention. Experienced qualitative researchers (LP,EG,LM,PH) familiarised themselves with the transcripts, then each transcript was coded in-vivo (i.e. codes derived directly from participants expressions about events, actions, values, beliefs or reflections) or from researchers’ understanding of participants’ expressions. Each transcript was coded by two researchers independently and differences in interpretation discussed. Coding was likely also informed by the prior development of guiding principles (Table 1) which may have primed researchers to ‘see’ issues (semantic meaning) and then consider the perceptions, experiences and contexts that may contribute to the construction of the explicitly reported issues (latent meaning). Multiple coders were used to draw on the team’s varied levels of experience with data collection and prior intervention development. This meant that a range of interpretations of the data, potential issues, their relative priority, and ways to address them were considered, to inform and optimise ongoing intervention development. A coding manual was developed to collate ideas from multiple team members and support the generation of themes. The coding manual was amended throughout data analysis and reapplied to transcripts to ensure all aspects of participants experiences had been captured. Coded data excerpts were collated in a spreadsheet, and analysed by systematically retrieving and comparing excerpts relating to each code. These coded data were then grouped into themes, for example ‘It’s not clear what to do’ included data coded as ‘easy to understand’, ‘not understood’ and ‘misundertanding the underlying message’. Data from each theme are summarised narratively.

## Results

Participant characteristics are shown in Table 2. Half of participants were living alone and most of those who were asked (i.e. Think aloud participants) rated their health as good to excellent, though they qualified this in relation to their own health status expectations or in comparison with acquaintances in their age-group. Some participants had a recent hospital stay or bereavement or received help with shopping and cooking. Only women were identified through snowballing.

**Table 2:** Characteristics of interview participants

	Think aloud: General practice n/16 (%)	Think aloud: Snowballing n/7 (%)	Process evaluation n/18 (%)
**Age range**			
65–74	3 (13)	1 (4)	8 (44)
75–84	7 (30)	5 (22)	9 (50)
85–94	5 (22)	1 (4)	1 (6)
Missing data	1 (4)	0	0
**Gender –**			
**Female**	9 (39)	7 (30)	11 (61)
**Male**	7 (30)	0 (0)	7 (39)
**Health conditions (self-report)**			
Cancer (not in current treatment)	2	0	2
Cardiovascular	7	3	8
Depression	1	4	2
Epilepsy	2	0	0
Eye conditions	1	1	0
Gastrointestinal	3	0	3
Leg ulcers	0	1	0
Musculoskeletal	7	6	2
Respiratory	6	0	6
Urinary tract	2	0	1
Missing data	1	1	0
**Self-rated health in last week**^a^			n/a
1–3 = Poor to very poor	1 (4)	1 (4)	
4 = Average	5 (22)	2 (9)	
5–7 = Good to excellent	10 (43)	4 (17)	
**Sheltered accommodation**	2 (9)	0	n/a
**Living alone**	7 (30)	7 (30)	8 (44)
**Recent hospital admission** **(last 6 months)**	2 (9)	2 (9)	2 (12)
**Bereavement in last year**	2 (9)	1 (4)	n/a
**Help to shop or cook**	6 (36)	2 (9)	n/a
**Indicators of low appetite / malnutrition risk**^b^	n/a		
MUST score = 1 or more			10 (56)
SNAQ score = 13 or less			12 (67)
BMI = 20 or less			6 (33)
Unintended weight loss in last 3 months			9 (50)
**Considered ‘at risk’ by family**		7 (100)	n/a

**Note:** ^a^Self-related health: “How would you rate your overall health during the past week? On a score of 1 to 7, where 1 is very poor and 7 is very good”

^b^ Self-report or nurse-measured *MUST* (Malnutrition Universal Screening Tool); *SNAQ* (Simplified Nutritional Appetite Questionnaire); BMI (weight/height^2^)

Four themes, generated from the data, are described below. The changes made to the intervention materials based on the data are summarised in Table 3. Participants were generally positive about the purpose and readability of the booklets, found them reassuring and liked the advice, tips and suggestions: *‘Yes, the booklets were so helpful…..and enjoyable reading anyway’[P140 PE].* However, this paper focuses predominantly on participants’ negative reactions, to illustrate the changes needed to ensure the intervention materials were optimally relevant and meaningful to older adults.

**Table 3:** Key changes made to ensure intervention materials were optimally meaningful to older adults

Purpose of change	Issues targeted	Examples and details
Strengthen perceived relevance of the booklets	Booklets not useful for those who already know about healthy eating and who are self-reliant	• Added emphasis that booklets provide new information on how eating needs change in older adulthood NOT general healthy eating advice • Added emphasis that suggestions in the booklets/goal setting can support continued independence and meeting eating needs • Added rationale that self-care ‘can help you to keep meeting your eating needs’
	Misunderstood purpose of booklets: interpreting them as purely promoting healthy eating rather than supporting eating for those with low appetite, weight loss or underweight	• Changed cover design to show varied high-energy food examples, including cake. • Entitled the main booklet ‘Meeting your eating needs as you get older’. • Clarified intended purpose on first page of each booklet (e.g. to address low appetite and unintended weight loss)
	Belief that weight loss is normal/inevitable and intervention not needed	• Clarified weight loss is not normal and highlighted the intervention can provide support to address reasons contributing to weight loss
	Booklets did not include enough information to suit individual needs and circumstances	• Expanded range of food suggestions to suit a variety of preferred and prescribed diets, following guidance from dietitians and nutritionists
Acknowledge and validate users’ prior experiences	Booklets did not acknowledge the range of challenges experienced by participants	• Added acknowledgement that it can be difficult to follow the advice and suggestions when appetite is low or you are unwell or in pain • Expanded content to address specific challenges experiences by participants e.g. changing taste sensations, reasons for finding cooking and shopping a chore, preparing food when in pain
	Booklets perceived as dictatorial and condescending	• The tone of the booklets was adjusted to offer suggestions to try
Encourage participants to seek support	Concern that nurses and doctors did not have enough time to discuss the booklets	• Added a ‘talk’ symbol to indicate key sections where discussion with a nurse or doctor would be most useful (e.g. making plans and goals). • Clarified that other people could also offer support (e.g. friend, family member, carer)
Use appropriate language	Confusion about key strategies for increasing nutritional intake	• Simplified key strategies and renamed to ‘adding tasty extras’ and ‘eating little and often’
	Specific phrases were off putting	• Replace aversive phrases with participants own language (e.g. ‘eating more’ replaced with ‘eating regularly’ or ‘adding tasty extras’, ‘snack’ replaced with ‘small bite’ • Removed reference to ‘full fat’ or ‘sugar’ and included these elements within a range of examples, e.g. adding cheese, fruit, jam or honey • Rationale and stories added to emphasise and model the benefits of regular eating and drinking

### Theme 1: who decides if nutritional support is needed?

While most participants described their beliefs about what constituted ‘good’ eating, or expertise in cooking, and self-reliance, there were some situations where support was welcomed. Some of these participants commented that nutritional support was not needed if they were already eating in a disciplined way or monitoring their weight to prevent weight-gain. Some also suggested that those in need of support would still not use the booklets, as they considered themselves set in their ways, accepting and self-managing any difficulties that they believed were inevitable in older age.

*Because you’ve got set things at an age you know, and the older you get I think the more you sort of think no, I am my own person and I am not going to be told what I…, you know, and to me that’s what that does[referring to booklet].[P333TA].*

Participants expressed contradictory beliefs about weight loss. Although most accepted that it is not good to lose too much weight, some were not convinced that it would be beneficial to gain weight, however little they weighed. Participants commented that it was normal to lose weight in older adulthood and often had an ideal weight in mind, if they felt better for having a low weight, were trying to lose weight or if they believed that lower weight makes it easier to get around or relieves their arthritis pain e.g. ‘*it’s a nice weight for me*’*[P012TA]*. Some wanted to gain weight but didn’t want to ‘overeat’ as they feared gaining fat rather than muscle.

*“Will I gain too much weight?” [reading from Main booklet] - that would be my concern, I lost weight when I was ill last year, but I lost muscle rather than fat which is infuriating, I still have fat*.[*P163TA]*.

Additionally, some did not agree that addressing loss of appetite or weight was a shared concern, stating that it was up to the individual to decide if they want to accept weight loss or not eat. For some this meant that the intervention materials were considered unnecessary, while others perceived the tone of the booklets as off-putting, dictating what one ‘should’ do, though one or two participants stated that they would prefer a more directive style.

*Information that’s in there was sort of interesting, but it wouldn’t have helped me specifically at any point…..I know I’ve got to eat and I get on with it [P611TA].*

There were some situations where support was welcomed, and some participants, particularly in the process evaluation group, expressed uncertainty about what to eat to when nutritional advice varied for their different co-morbidities. Participants from both groups commented that the booklets needed more tailoring to their specific problems, for example requesting information about foods that provide high energy or contain protein or carbohydrate, to better address loss of strength and feeling tired. A few wanted clarification about what they ‘should’ be eating, if they found advice delivered via broadcast media unhelpful.

*Because you can look at magazines, or like my granddaughter went online, but it’s so confusing, you don’t know, there’s nothing to tell you what’s best for your particular case or your age, as it were.[P166PE].*

Some therefore welcomed GP or nurse support to assist them in using the booklets; to explain why they are useful, start a conversation about nutritional concerns and encourage them to enact the suggestions. However, some participants expressed concerns about adding to healthcare professionals’ workloads.

*GPs are overworked aren’t they and very busy and haven’t got a lot of time - if she’d sort of said well go to the practice nurse and discuss the booklet with them, then I think it would be useful.[P39TA].*

### Theme 2: what does eating better mean?

In early Think aloud sessions, participants understood the broad aim of the booklets in supporting beneficial eating habits among those with appetite or weight loss, but did not easily recognise the range of health goals that the booklets were designed to support. Some also wondered whether the booklets would support weight loss as well as weight gain attempts, while for some, the phrase ‘eating and drinking more’ risked encouraging inappropriate weight-gain.

*If they want to put on weight or if they want to…, I suppose if you ate like this tells you… if you wanted to lose weight it would help as well would it?[P39TA].*

*‘Common reasons for wanting to eat and drink more’, well I mean so many people do it nowadays and they just get absolutely obese.[P223TA].*

Throughout the booklets, two key strategies for enhancing nutritional intake were promoted: eating more at each meal (e.g. ‘give your food and drink a boost’) and eating between meals (e.g. ‘snacking’ or ‘top up food’). However, participants in Think aloud sessions commented that they were not always sure what the different phrases were encouraging them to do and some did not see why these suggestions were beneficial, or stated that more specific, clear detail was needed about what constituted ‘better eating’.

*Well, what does it mean saying ‘eat or drink better’? Eat and drink better things or better foods or to be more, you know sort of plan, have, eat when you should be eating or not, rather than not eating?[P33TA].*

Initially, the word ‘snack’ and the concept of snacking was used to convey the idea that eating small amounts between meals can provide a steady flow of energy and increase food intake at times of reduced appetite. There was divided opinion about whether snacking was considered to be good or bad. While some participants stated that it was good to highlight that snacking was not forbidden, many found ‘snacking’ aversive e.g. ‘*I cannot go along with the snacking*’*[P513TA]*. Various reasons were given, including not being brought up to snack, or seeing snacking as an improper way of eating or forming a bad habit, which was associated with people who ‘*probably didn’t know what to do*’[*P593TA*]. Some expressed the belief that snacking reduces hunger at mealtimes, or simply stated:*‘we don’t eat in between meals’[P14TA].*

Some participants did eat between meals, for example having something to eat with a cup of tea, eating chocolate or fruit when they ‘fancy’ it, or eating something if they are hungry and reducing the size of their subsequent meal, but did not perceive this as snacking.

*I wouldn’t call it snacks, I would call these, umm ‘my little bit’, you know, if I have my Brioche roll and my cheese.[P002TA].*

In early drafts, the booklets encouraged the inclusion of high energy food in the diet, particularly full-fat and sugary products to ‘give a boost’, but participants’ awareness of current public health messages appeared to deter them from trying out such suggestions. For example, some participants were strongly opposed to full-fat or sugar, referring to them as things that you ‘*spend all your life trying to avoid’ [P223TA]*, and some disliked the taste or texture of full-fat dairy products.

*No, I don’t believe in that [using sugar], I don’t like sugar in teas or coffees or anything… No, I don’t have full-fat milk.[P111TA].*

Other participants were happy to use full-fat milk and sugar-sweetened products, if they were already doing this, they accepted the rationale in the booklets, a GP had also suggested this, or they believed in ‘everything in moderation’.

*That’s interesting, because so many people think, ‘oh you must never have sugar’, and of course you* should *have so much sugar.[P64PE].*

### Theme 3: how will eating better help ME?

Prominent among participants’ comments was the extent to which the booklets and other materials were personally relevant to their needs, preferences, circumstances and habits. Many stated that their weight was stable or had not weighed themselves recently, and these participants commented that they did not identify with early drafts of the booklets because the focus was perceived to be on addressing excessive weight loss. Participants instead suggested the booklets would be useful for others, who are frailer, less capable, have more problems, or don’t already use the strategies listed.

*They were informative – but not something I would bother with myself, because as I say, I tend to eat what I want.[P32PE].*

Participants who had experienced regaining weight after a decline, or who were physically active or still working, appeared more convinced by the rationale that eating can provide energy for everyday activities or to support recovery. Additionally, some process evaluation participants commented that they did or would have found the booklets useful directly after hospitalisation or bereavement, but once things started to improve, they were no longer needed.

*You see at the moment I’m doing alright. I wish I’d known about this sooner…..[the booklet] about the supplements…..Hmm, now that would have been very interesting when I came out of hospital.[P166PE].*

Participants did not all agree that the ‘common reasons for wanting to eat or drink more’ were relevant. For example, getting better more quickly when ill would depend on what was wrong with you, with one participant commenting that antibiotics, rather than eating, make you well. The suggestion that movement can ease pain also appeared less convincing if participants had not previously experienced this for themselves.

*Well you’re saying there …[try] eating … when you’re in pain, because it will give you energy to continue moving, and moving can help ease the pain. Does it?... I would have thought it had to be, I don’t know, worded a bit differently...[P333TA].*

Some participants described specific foods that they were advised to avoid due to specific health conditions, or that they found indigestible, such as bread, and many stated that their sense of taste or smell had deteriorated or intensified, making once-loved foods intolerable. Some described ways of eating that helped them manage taste problems.

*I eat more sweets, and I never ate sweets before, but I do now, because of the horrible taste I get in my mouth [from prescribed drugs].[P001TA].*

### Theme 4: can I follow the tips and strategies?

In early drafts, participants appeared to be unconvinced by some of the most important messages the booklets were designed to convey. The goal of eating little and often, initially conveyed as eating three meals a day plus snacks or nine small meals elicited a strong negative response. Most participants asserted that having multiple ‘small meals’ a day sounded over-facing, would take up too much time or would be an inappropriate behaviour.

*[Reading from booklet] “Three big meals and three snacks a day”. Stone me, you’ve got to be joking![P213TA].*

Participants also commented that they found the idea of eating bigger meals off-putting and effortful or stated that both small and large plates full of food deterred them from eating.

*So ‘bigger helpings’, no I don’t think so. ‘Use a bigger plate’, my plate’s about that big now [shows a small size with hands], so, no. When we go [out] I’ll only have a small plate…[so] you will only get a small portion.[P007TA].*

Opinion was divided about the relevance and usefulness of goal-setting. A few participants stated they would use the goal planning chart, if they remembered, but some commented that while the example goals were relevant and useful, they would not necessarily try it themselves. Others commented that they had never needed goals in the past, or could not see how the example goals would fit into their unpredictable lifestyle.

*I: OK, so the goal-planning booklet, was that not helpful?*

*P: Umm, if I’m honest, no, because my life is chaotic.[P74PE].*

Participants agreed that not wanting to prepare food or cook could get in the way of eating. Some commented that it would be useful if the booklets acknowledged different reasons for not cooking, such as disliking cooking, enjoying cooking but not being able to cook due to physical frailty or pain or finding recipes too challenging. Participants also shared strategies and tips that had worked for them, such as batch cooking and freezing favourite dinners to reheat when food preparation was challenging.

*‘I don’t like cooking.’ ‘I don’t know how to cook.’ Well I do know how to cook, but I don’t like to, so what shall I put there?[P005TA].*

## Discussion

This paper outlines how we applied iterative qualitative techniques to systematically develop an intervention (Eat well, feel well, stay well), first applying evidence from our mixed methods synthesis [23] and then optimising the intervention using participants’ feedback from a Think aloud study and a process evaluation study. While it remains to be tested, this approach gives confidence that the intervention may now be somewhat more likely to be acceptable and meaningful to target users [44]. Although we aimed to identify beliefs or contextual issues that undermined older adults’ engagement with a specific intervention to address risk of malnutrition, these findings may also have wider implications for designing other types of interventions, particularly for older adults.

### Improving personal relevance

Despite being selected because they had health or social conditions that are associated with increased risk for malnutrition, participants generally stated that the booklets would be useful for others but were not relevant for themselves. This highlights that key health beliefs (e.g. perceived susceptibility (Health Belief Model [45]) or risk perception (Health Action Process Approach [46]) were not sufficiently addressed by the intervention). Low perceptions of susceptibility may reflect a current public misperception about malnutrition risk being evident only when individuals appear thin or frail, or a reluctance to see oneself as ‘at risk’. Although we avoided using the term ‘malnutrition’ in the booklets, due to aversive connotations [23, 30], and participants endorsed some concepts and suggestions, lack of perceived personal relevance could still hinder their adoption, as found in previous studies (e.g. Yardley 2012 [47]). Tunnelling, tailoring or personalisation have been used to enhance the perceived personal relevance of behavioural intervention content (e.g. Van Velsen 2019 [48]), so as to encourage attention to, and persuasion to accept, health messages [49]. In order to improve personal relevance in response to participants’ comments, we clarified the purpose and scope of the intervention on the initial page of each booklet and re-designed the covers. We emphasised that the advice was not only to support people with obvious signs of malnourishment, but also for those with (sometimes intermittent) risk indicators such as low appetite, unintended weight loss, or lower than usual weight for their height and age-group. To aid tunnelling, we signposted booklets that addressed specific needs, and increased the variety of food options and suggestions so that users could self-tailor by choosing which were most useful and preferable to themselves [50]. Tailored feedback has been previously found to be effective in print-based behaviour change interventions [51], so was supported by adding speech bubbles to indicate sections that users might find it helpful to discuss at nurse appointments. The final booklet versions give a variety of food ideas including options to help people avoid the foods that they need to, if they are on a special diet, and recommend that people on a special diet should speak with their nurse or GP. Additional booklets addressing malnutrition risk in specific health conditions could be developed, but in this study, participants with various health conditions appeared to be aware of current eating advice for their specific condition and so self-tailoring may be a good option. When developing interventions, it is important to explore the extent to which users, in this case older adults, are open to change, resist the temptation to design interventions that meet researchers’ or clinicians’ priorities rather than older adults’ needs and expectations, and to address potential causes of disengagement by first hearing what is important to users.

### Positive messages about eating to address age-related decline

One of the current project’s guiding principles was to use a positive tone throughout, as previous research suggests that older adults are more likely to attend and respond to positively framed health messages pointing to a positive outcome, for example adopting specific dietary practices can help to maintain health or prevent deterioration [52–55]. Adopting a positive tone enabled a focus on easily achievable tips, such as easy to prepare food, but also to normalise difficulties and offer strategies to address them. However, extending previous research, participants in this study were reluctant to accept concepts within the booklets if they perceived the tone of information or suggestions to be pressurising or patronising, even when positively framed. When sharing expert knowledge, conveying information too simply may inadvertently reinforce age-related stereotypes and negatively influence self-care [56]. The implication is that giving positive messages about eating to address age-related decline is not enough on its own. By providing suggestions that ‘you may like to try’, including examples that worked for participants, and addressing users as ‘you’ rather than ‘we’, we aimed to offer options without pressure or condescension. Whether these measures are sufficient to encourage older adults at risk of malnutrition to adopt some of the suggested behaviours now needs to be tested in practice. When developing interventions for older adults it is important to challenge our own assumptions about optimal communication methods and to check how messages are perceived by target users and Patient and Public Involvement collaborators.

### Making key messages meaningful

In this study, paying attention to participants’ responses to specific words and their connotations initiated changes to make key messages more meaningful and persuasive. All the changes we made (Table 3) were designed to ensure that the intervention was optimally meaningful to older adults. Participants’ strong negative response to messages about eating bigger meals and frequent meals or snacks each day significantly influenced intervention optimisation. Participants’ preference for smaller portions concurs with previous qualitative research [52] while the suggested strategy of eating little and often may combat decreasing metabolic rate during ageing by reducing portion sizes and encouraging more regular nutritional intake [57]. At the same time, it was necessary to support greater energy intake for older adults at risk of malnutrition through increased meal size. Adjusting the wording used to convey these messages, e.g. ‘adding tasty extras to your usual plate’ instead of ‘bigger meals’ seemed to make this strategy more acceptable by appealing to desire and enjoyment rather than hunger, which can be impaired in older age, particularly among those who are undernourished [58]. In response to participants’ requests, and in line with evidence of the protective effects of protein intake against physical decline from the PRevention Of Malnutrition In Senior Subjects in the EU (PROMISS) consortium (e.g. Mendonca et al. [59]), our ‘Food list’ highlighted protein-rich foods, and we included protein in most meal and snack suggestions throughout the booklets.

It was also important to consider other health messages and ensure that suggestions were compatible with these. We needed to address misconceptions stemming from general public health messages e.g. encouraging low fat and sugar intake, that may not necessarily reflect changing needs as we get older. While snacking as a concept was disliked by participants in the present study, ideas for snacks e.g. rice pudding with raspberries were liked, and participants mentioned many foods that they would eat between meals. Extending previous research showing that specific words and phrases can trigger negative connotations about eating behaviours [60], this study found that users might be open to eating between meals if the wording was changed to ‘eating and drinking regularly throughout the day’ and having ‘small bites’ or ‘little extras’, and a rationale provided. When developing interventions for older adults it is important to identify any widely-held beliefs or specific words and phrases that may undermine key messages in the intervention. It is also important to avoid using words and phrases that are aversive or evoke a feeling of being dictated to, which risks rejection of suggested activities.

### Strengths and limitations

Strengths of the present study include the rapid incorporation of intervention changes from interviews; using a table of changes to transparently capture, agree and prioritise changes aided by our evidence- and context-based guiding principles; and expert opinion from our clinical and public and patient representatives. We also confirmed that it is crucial to carry out iterative changes after a prototype has been developed: the prototype addressed key issues identified during a systematic review, exploratory qualitative work and the use of guiding principles (Table 3), but it was clear from the Think aloud sessions that the issues had not been addressed in a way that was meaningful to participants. The Think aloud results allowed us to adjust the prototype to make it more meaningful. The smaller number of process evaluation interviews quotes in the results section reflects that the purpose of the process evaluation interviews went beyond exploring how the booklets were received. Nevertheless, insights from these feasibility study participants with objectively assessed risk factors for malnutrition in addition to characteristics associated with greater risk (i.e. those included in the Think aloud sessions), were crucial in allowing us to make sure that the booklets were more meaningful.

We included men and women of a range of ages with different health and social conditions known to increase risk of malnutrition. However, we may have missed those who are most ‘at risk’, who may have greater challenges to participation. We did not collect ethnicity, education or income data, so were not able to report on these characteristics. We asked most participants to ‘Think aloud’ without time to digest content of booklets in advance, perhaps reducing cognition/reflection on the content, especially among older adults who may have some degree of cognitive decline. However, we did gain an indication of how booklets might work when introduced by a healthcare practitioner, so that we could recommend that nurse and patient talk through the key parts of the booklets.

The optimised ‘Eat well, feel well, stay well’ intervention outlined in this paper now needs to be tested in the setting in which it is designed to be used. While improvements made to the booklets were well-received once issues raised by participants had been addressed, further issues may be identified in a cluster randomised trial (currently ongoing) and further adaptations may still be necessary.

## Conclusions

Iterative qualitative methods allowed us to identify specific ways in which older adults responded to key messages, strategies and tips suggested in the booklets and other materials we developed to address malnutrition risk. Had we not explored older adults’ perspectives we would not have uncovered the widely-held beliefs, specific words and phrases that supported or undermined key messages. Hearing what was important to participants allowed us to make small but crucial adjustments, without which the booklets would have been insufficiently meaningful, persuasive and relevant, with older adults potentially being less engaged with the intervention. Further replication and elaboration of the methods described, particularly making efficient use of Tables of changes, or other ways to rapidly iterate intervention improvements while remaining transparent and robust, would be helpful. We are now testing the impact of the changes made to the intervention in a RCT.

## Supplementary Information

**Additional file 1.** Mapping booklet contents to guiding principles: Main, goal and optional booklets (pdf).

**Additional file 2.** Interview guides (pdf).

## Data Availability

The datasets supporting the conclusions of this article are included within the article and its additional files.

## Abbreviations

C19: Coronavirus covid19
COREQ: Consolidated criteria for reporting qualitative research
MNA: Mini nutritional assessment
MUST: Malnutrition universal screening tool
PBA: Person-based approach
PPI: Public and patient involvement
RCT: Randomised controlled trial
SNAQ: Simplified nutritional appetite questionnaire
TOC: Table of changes
