# Supplementary material for: Optimising an intervention to support home-living older adults at risk of malnutrition: a qualitative study
Source: BMC Fam Pract. 2021 Nov 11;22:219. doi: 10.1186/s12875-021-01572-z (PMC8580738; doi:10.1186/s12875-021-01572-z)
Supplement: Supplementary file 1 — Additional file 1. Mapping booklet contents to guiding principles: Main, goal and optional booklets (pdf). [file 12875_2021_1572_MOESM1_ESM.pdf]

Additional file 1. Mapping booklet contents to guiding principles: Main, goal and optional booklets

**Guiding principles (design objectives with intervention features)**	***Main booklet***	***Goal booklet***	***Booklet 1: I don't fancy food or drink***	***Booklet 2: I have problems chewing or swallowing***	***Booklet 3: Cooking or shopping feel like a chore***	***Booklet 4: I don't enjoy eating on my own***
**Design objective 1:** Motivate engagement with lifestyle change						
**Intervention feature:** Credible evidence-based rationale	**Key Message**<br>Eating needs change as you get older.<br>Particularly if you have low appetite, lost weight without meaning to or weigh less than usual for age and height<br><br>**Key Message**<br>Eating and drinking regularly throughout the day gives energy and strength to keep doing all you want to	**Key Message**<br>Goal-setting booklet can help you decide how to keep meeting eating needs, and track progress<br><br>**Rationale**<br>People can find it useful to set a goal to do something new or keep up their current habits	**Key message** Important to keep eating even if you don't feel like it<br>**Rationale**<br>Gives body the goodness and energy needed to work well, stay healthy and independent<br><br>**Key Message**<br>Need to drink plenty even if it means peeing more<br>**Rationale**<br>Flushes out infections, lower blood pressure, improve sleep<br>**Did you know**<br>Pee is colour of straw if you're drinking enough<br><br>**Key Message**<br>Eating and drinking regularly throughout the day helps body deal with waste products	**Key Message**<br>Eating regularly throughout the day is good<br>**Rationale**<br>Eating regularly gives you the goodness and energy needed<br><br>**Rationale**<br>Tips in this booklet can help you to keep eating the food you like<br><br>**Rationale**<br>Evidence-based advice and has worked for other people with problems chewing or swallowing	**Key Message**<br>There are lots of ways to make shopping and cooking easier and take less time, boosting appetite and making eating more enjoyable<br><br>**Did you know**<br>Pre-made meals can be good quality	**Key Message**<br>There are lots of ways to make eating on your own more enjoyable<br><br>**Rationale**<br>Eating less can mean you don't have enough energy, then feel tired, cold and mood affected

	**Key Message**<br>Drinking is important too<br><br>**Rationale**<br>Evidence-based advice and has worked for other older adults (helps you to stay fit, healthy and independent)		**Rationale**<br>Fibre helps push waste through your digestive system, so helps make going to the toilet easier<br><br>**Key Message**<br>Eat regularly even when feeling unwell<br>**Rationale**<br>Helps the body fight illness / infection<br><br>**Key message**<br>Food and drink can help with pain<br>**Rationale**<br>It helps you have energy to move about, and moving can help with some pain			
**Intervention features:**<br>Dispel myth that decline in appetite and eating, and weight loss are normal and inevitable in older age;<br>Clarify that everyone can be at risk of malnutrition;	Advice may not be quite the same as you've been told before, because eating needs change as we get older (e.g. low fat, low sugar may be less appropriate)<br><br>Changes happen in older adulthood can affect		It's normal to lose appetite when not feeling well<br><br>Developing habits –means you can get used to eating / not eating	Outline common problems and common causes	Dispel myths about some foods, e.g. frozen fruit and veg is as healthy as fresh, ready (pre-prepared) meals can be healthy	It can be easy to skip meals when you eat alone, especially if you're used to eating with someone

Outline that mainstream public health messages may be less appropriate for those with low appetite or unintended weight loss	appetite, with examples<br><br>Common questions to highlight risk factors e.g. *"Isn't it normal to lose weight when you get older?"*					
**Intervention feature**<br>Demonstrate empathy and acknowledge real barriers to change, including awareness of age-related decline	Can be difficult to eat without much appetite	Things can get in the way and it is normal to have ups and downs - then you can try again	Sometimes people don't feel hungry, for many different reasons (common ones are listed)<br><br>It's normal for sense of taste to change<br><br>Pain takes up energy and attention, so makes preparing food, and eating and drinking, harder	It's not always easy to eat when you have chewing or swallowing difficulties<br><br>It can take longer to eat, can be uncomfortable and worrying	Can be hard to decide what to eat and shopping or cooking can feel like a chore<br><br>Examples of what might stop you wanting to cook or shop	It's not unusual to eat less / skip meals if you often eat on own, especially if used to eating with another person
**Design objective 2:** Promote self-efficacy to manage malnutrition risk, and overcome barriers to eating well						
**Intervention features:**<br>Positive tone to encourage beliefs about being able to overcome barriers;<br>Align behavioural advice and support with	Booklet has easy tips to help meet your eating needs even when you don't feel like eating		You can eat and drink even if you don't feel hungry	You can still eat even if you have problems chewing or swallowing	Eating well doesn't have to be expensive	

need to be 'well' and independent						
**Intervention feature:**<br>Provide examples of small, easy to enact lifestyle-compatible changes<br><br>(N.B. Easy tips that experts recommend and have worked for others. All Tips are followed by food or other examples)	**Main tip 1**<br>Eat little and often (For low appetite, aversion to large meals, when skipping meals)<br><br>**Main tip 2**<br>Add tasty extras to your usual plate<br><br>**Tasty ideas**	Example goals relating to Main tip 1 and Main tip 2, with food ideas<br><br>**If…then…. examples**, using cues to eat little and often, and to add tasty extras to my usual plate	Don't fancy food: Eat a small bite at set times (little and often); add little extras (to your usual plate); move before eating and move about more during the day; Have liquid-based foods or other drinks instead of tea and coffee<br><br>Pain: ways to make food and drink easier to prepare<br><br>Hydration: drink in between 'meals' to feel less full;<br><br>Breathless: choose soft moist food that is easy to swallow<br><br>Dry mouth: brush teeth and floss before eating, suck frozen fruit to make saliva<br><br>Taste changes: foods / drinks that oppose bitterness, sweetness, metallic taste, no taste; follow medication with a tasty treat<br><br>Worries about toile: drink and eat earlier in the day; ask GP to refer for advice / equipment	General mouth care: practise chewing without food in mouth, keep teeth clean, visit dentist regularly<br><br>Mealtime routines: eat little and often, eat slowly and chew well before swallowing, sip water after each bite, eat a while before bedtime<br><br>Make the food itself easier to eat: if difficult to chew: cut into small pieces and take small bites, blend slightly with food process or fork, eat thicker liquids e.g creamy soup or slightly blended chunky soup<br><br>Make the food itself easier to eat, if too dry: moist instead of	Ways to save money at supermarket, e.g. special offers, own brands, money-off coupons<br><br>Making shopping easier: use the freezer, have home deliveries, stock the cupboard, order ready-made food	Ways to help you look forward to eating, listen to music, radio, TV, read; set the table, mindful eating; visual aids, e.g. eat colourful food, garnishes; eat with others e.g. friends, or visit cafes

			Constipation: eat food with fibre, pulses, citrus, water or juice, take your time and relax on the toilet, and go when you need to<br><br>Can't face food at all: ask GP or pharmacist for supplement	sticky food, moisten dry food e.g. with gravy, suck ice cubes to increase saliva		
**Intervention feature:** Allow self-tailoring to address personal barriers	What gets in the way of eating or drinking regularly, leading to choosing other booklets, with talk symbol<br><br>Common reasons for wanting to eat and drink regularly	Two key suggested goals / plans to address different preferences, plus self-select goals / plans<br><br>Food examples: ways to help with specific problems	Separate sections for specific personal barriers: just not hungry; loss of taste; don't want to go to loo; constipation; pain; don't feel well	Separate sections for general mouth care; mealtime routines; making the food itself easier to eat	Food examples for people who prefer larger or smaller meals Quick and easy meals<br><br>Suggestions for shopping / eating on a budget; home deliveries	Tips and examples both for people who like eating on their own and those who do not
**Intervention features:** Offer longitudinal motivational support; Support motivation in-the-moment		Highlights that keeping track can help you stay motivated and spot problems early<br><br>Self-monitoring, with batteries analogy<br><br>Self-monitoring with a progress chart	Suggests ways of making food more tasty<br><br>Highlights that preparing and cooking food can distract from pain	Highlights that you can still eat even if you have problems chewing or swallowing	Visual cues in pictures of varied and tasty food<br><br>Variety of easy food ideas	Offers suggestions to make eating more enjoyable e.g. making mealtime more ambient by setting the table

**Intervention feature:**<br>Support personal goal and action planning	Suggest that older adulthood can be a useful time to think about what you eat and drink<br><br>Refer to goal-setting booklet	**Talk symbol:** You can talk about your goals and progress with your nurse, GP, carer, friend or family member. They may be able to help you think about what you choose to do<br><br>Suggests thinking about actual foods and drinks as well as your goal<br><br>Suggest that it can be helpful to plan how and when you will eat and drink	Suggests making tips from this booklet into goals, and tracking them	**Talk symbol**<br>To suggest talking about goals and progress with nurse, GP, carer, friend or family member	**Activities**<br>Tick the boxes to mark tips you'd like to try	
**Intervention feature:**<br>Provide stories to model successful ways to overcome barriers	**Story (Pat):** eating little and often<br>**Story (Chris):** adding tasty extras to your usual plate	**Story (Sam):** I used reminders to help me			**Story (Charlie):** going to shop with shopping list	
**Intervention feature**<br>Signpost **local resources** and sources of support (other	Refers to other booklets for more ideas	Refers to other booklets for more ideas	Refers to 'My personal goals' booklet, to turn tips into goals / plans<br><br>Useful websites	Tips should be discussed with GP or nurse. Always follow GP or nurse advice	Refers to other booklets for more ideas (Food list, friends, recipe cards, magazines,	Refers to food list and 'Cooking or shopping feel like a chore'

booklets, healthcare staff at the GP surgery, community resources)	Who to ask if you have a question or concern	Who to ask if you have a question or concern<br><br>Use the goal cards to help you meet your goals	Ask GP surgery if questions or concerns	If you continue to have problems, talk to GP<br><br>Who to ask if you have a question or concern	internet, local library)	Refers to support from national organisations with helplines and befriending services
**Design objective 3:** Promote support and autonomy for choosing lifestyle changes that harness personally relevant motivations						
**Intervention features:**<br>Present rationale for lifestyle change with a non-directive tone;<br>Present behavioural suggestions as options to try;<br>Invite expression of preferences;<br>Acknowledge and validate existing knowledge and experience before introducing new information and advice	Common questions section highlights risk factors using positive tone e.g. addressing common views about weight loss and gain, and appetite changes during ageing<br><br>Lists possible reasons for wanting to eat well, with tick boxes for self-selection	Suggests optional goal planning and tracking	Suggests options to help with specific common reasons for not feeling like eating, including food preparation and eating behaviours, but also behaviours related to e.g. pain management, toilet habits	Suggests options to make eating easier with chewing and swallowing. Includes functional options e.g. ways to strengthen jaw muscles to make chewing easier	Quick and tasty ideas for small and larger bites, taking 5-15 mins to prepare or ½ hour in oven<br><br>Tasty ideas for adding extras to favourite dish<br><br>Tasty ideas of bites for any time of day	The easy tips can make eating for one more enjoyable so that you look forward to cooking and eating, have energy to do things you enjoy
