# Supplementary material for: Optimising an intervention to support home-living older adults at risk of malnutrition: a qualitative study
Source: BMC Fam Pract. 2021 Nov 11;22:219. doi: 10.1186/s12875-021-01572-z (PMC8580738; doi:10.1186/s12875-021-01572-z)
Supplement: Supplementary file 2 — Additional file 2. Interview guides (pdf). [file 12875_2021_1572_MOESM2_ESM.pdf]

Additional file 2: Interview guides for 'Think Aloud' and 'Process Evaluation'

Eat well feel well: a study about people's appetite and eating patterns

Interview guide: Think aloud interviews with patients

In this study we will ask for patient's views about materials (eg. booklets) that we have developed. The aim of the materials is to encourage people to eat and feel well. The materials will form part of a screen and treat intervention for people with low appetite or eating and drinking patterns that may affect their health.

Further questions may be added as the intervention is developed. This will ensure that the questions are relevant and easily understood by participants. It will also allow us to include questions based on comments by participants in early interviews.

Explaining the task to participants

In this study we are asking for people's views about some materials / booklets that we have made. The booklets are about eating and feeling well. They are designed for people of 65 and over who have health issues or live alone or have had a recent stay in hospital.

We are interested in your views about this version of the materials / booklets. We are not asking you to do what it says in the booklet.

Please can you pretend I'm not here and look at the materials / booklets and talk out loud. Just say out loud what you are doing, what you think and feel about each page and what is on each page. If you have any questions, try to imagine I'm not here and talk out loud as you look for an answer.

After you have finished looking at the materials / booklet, I would like to ask you about your overall views of the materials / booklet(s).

We can take a break at any time you like, please just let know and I can pause the recording. We can also stop the chat at any time if you want to.

Is there anything you would like to ask me?

Are you happy if I start recording now?

Start: Please can you look at the materials / booklet(s) and start to talk out loud.

Possible prompts for the researcher to use during Think Aloud

1. Can I ask what are your first impressions of this page?
2. Can you tell me what you are thinking now?
3. Can you explain what made you choose that option?
4. Can you say what you think about this suggestion / activity?
5. Can you tell me a bit more about why you think that?
6. What is it you like about that?
7. What is it you don't like about that?

End: Thank you – is there anything else you would like to say?

Post Think Aloud questions

Start: I'm really interested in how you are finding the materials / booklet overall…..

1. Can you tell me, overall, how you found the materials / booklet(s)?
2. Can you tell me about anything you thought was good about the materials / booklet(s)?
3. Can you tell me anything about the materials / booklet(s) that you were less keen on?
4. Is there anything you think should be changed?
5. Can you tell me about anything else that might be useful for you?
6. Can you show me which parts would be most relevant to you?
7. Can you tell me how you would find it if your GP or practice nurse asked you to use the materials / booklet(s)?
8. If you were following the materials / booklet(s), can you tell me if you would try any of the suggestions? Can you tell me a bit about why?
9. Could you tell me about anything that would make it easier to follow the suggestions shown in the materials / booklet(s)?
10. I'm interested to hear about anything that would make it difficult to follow the suggestions shown in the materials / booklet(s). Can you tell me a bit about this?
11. When people use the materials / booklet(s) they may be able to have help to go through them. This might be in person, by telephone or by email. Could you tell me a bit about what might suit you best?
12. This support could be given to you by a nurse or someone else. Could you tell me a bit about who you would prefer to give you support and why?

Optimising the meaningfulness of an intervention to support home-living older adults at risk of malnutrition: A qualitative study

Interview guide: feasibility study process evaluation

This interview guide is designed to ask about patients' experience of taking part in the Eat well, feel well, stay well feasibility study. This will include asking patients about their appetite and eating patterns and habits, their views about booklets and other materials we have developed, and how it was taking part in the study. Patients' views will help us to improve the materials, and how malnutrition screen and treat policies are delivered in doctors' surgeries, which will then be tested in a randomised controlled trial.

The interview guide will evolve over time based on responses from early interviews, to ensure that questions are relevant to, and understood by participants.

**General open questions**	**Possible probing / prompting questions**<br>(to use in response to participants' comments)
1. I'm interested to hear about your appetite – could you tell me how you are finding this at the moment?	• Could you describe what you like to eat and drink?<br>• Could you tell me about the sort of things that affect your appetite?<br>• Could you describe times and places when you tend to feel hungry or thirsty?<br>• When you feel hungry or thirsty, can you talk me through what you do?

**Questions about being in the study**	**Probing questions**
1. I'd be really interested in how you found the information you were sent about this study. Could you tell me about this?	• Can you talk me through the different information you had about the study?<br>• Can you tell me what you thought of the information you were sent – both positive and negative?
2. I'm very interested to find out what it was like being in the study. Can you tell me a bit about that?	• Could you tell me what made you want to take part?<br>• Can you talk me through what you have done in the study?
3. I'm interested to hear how you found it filling in the list of questions *like this one (show example)* that you were sent. Could you tell me about this?	• What did you think of the questions?<br>• What did you like about the question list?<br>• What did you like less about it?<br>• We are thinking of changing some of the questions – could you take a look at the new questions and let me know what you think of them?<br>• How easy would you find it to answer the new questions?<br>• How relevant are the new questions to you?<br>• Which of these are most important for you?<br>• What other things are important to you? E.g. improve appetite? Quality of nutrition in what you eat? Not getting frail? Getting less infections? Anything else?
4. I'm really interested to hear about your appointment(s) with the nurse / doctor. Could you tell me about this?	• Can you tell me about any appointments you have had with the nurse / doctor as part of the study?<br>• If you had a phone appointment with the nurse / doctor, can you tell me what that was like?

	• Can you talk me through what happened at the appointment(s)?<br>• Did the nurse/doctor ask about your appetite and eating patterns? If so, how did you find this?<br>• Will you have any more appointments?<br>• Did you make the appointment with the nurse or did the nurse phone you?
5. I think you were given a form to fill out about your general health like this one (show example). I'd be interested to hear how you found that*?*	• Can you tell me about anything else that might be useful to add to this?<br>• Can you show me which parts are most relevant to you?
6. I'm interested in whether you were given or tried special drinks from your doctor. Could you tell me a bit about this?	• If yes, can you describe how this came about?<br>• If no, have you ever had special drinks?<br>• Can you tell me how you find / found the special drinks?<br>• I'm interested to know what your appetite is / was like while you are / were taking special drinks. Can you tell me about this?<br>• I'm interested to know what your eating patterns are / were like while you are / were taking special drinks. Can you tell me about this?<br>• What about drinks you can buy from the pharmacy, such as Complan? Have you ever tried these? Can you tell me about this?
7. Only for people who offered to give urine and / or blood samples as part of the study	• If you gave some urine samples, can you talk me through how you found that?<br>• If you gave some blood spot samples, can you talk me through how you found that?<br>• If you didn't, could you say why?
8. Is there anything you'd like to change about your eating habits?	
9. Is there any advice about eating that you would find useful?	
10. Have you had an appointment with a different nurse to measure your grip strength?	• If so, how did you find it?

**Questions about the booklets / materials**	**Probing questions**
1. I'm really interested I'm interested in any booklets and other materials that the nurse / doctor gave you. Can you tell me a bit about this?	• Can you tell me which booklet(s) and other materials you had / have?<br>• Can you talk me through how you decided which booklets to take?
2. Can you tell me, overall, how you found the booklet(s)?	• Have you read any of them? If so, what did you think?

	• Have you used any of the tips in the booklets? If so, how has that gone?<br>• Have you used any of the tick boxes in the booklets?
3. Can you tell me about anything you thought was good about the materials / booklet(s)?	•
4. Can you tell me anything about the materials / booklet(s) that you were less keen on?	
5. Is there anything you think should be changed?	
6. Can you tell me about anything else that might be useful for you?	
7. Can you show me which parts are most relevant to you?	

Thank you: is there anything else you would like to say?
